# Intramedullary Screws versus Kirschner Wires for Metacarpal Fixation, Functional, and Patient-Related Outcomes

**DOI:** 10.1055/s-0038-1637002

**Published:** 2018-03-09

**Authors:** Jose Couceiro, Higinio Ayala, Manuel Sanchez, Maria de Los Angeles De la Red, Olga Velez, Fernando Del Canto

**Affiliations:** 1 Orthopedics Department, Hand surgery Unit, Hospital Marques de Valdecilla, Santander, Ca, Spain

**Keywords:** metacarpal, fracture, screws, wires, function

## Abstract

**Purpose** The purpose of our study is to compare the intramedullary fixation of metacarpal fractures with cannulated headless screws and antegrade Kirschner wires in terms of final total active motion, grip strength, patient-related outcomes, need for casting, and return to work times.

**Methods** The authors performed a retrospective review of the hospital records. Thirty fractures were included in the study, 19 in the screw fixation group, and 11 in the Kirschner wire group. Grip strength, and total active motion, was measured at the latest follow-up for both the injured and contralateral hand. Pain was measured on the visual analog scale. Patients were requested to fill a Quick disabilities of the arm and hand score (DASH) questionnaire at the latest follow-up. Satisfaction was measured on a scale from 0 to 10. The time to return to work was quantified from the accident to the point when the patient was back to active duty. Postoperative casting time was also quantified.

**Results** The authors did not find any differences between the two groups in total active motion, grip strength, pain, satisfaction, or Quick DASH scores. We did find a difference in the return to work and casting times; these appeared to be shorter in the screw group.

**Conclusion** Due to the small number of cases, we have been unable to clearly conclude that there were any benefits in the application of one particular technique when compared with the other.

Extra-articular metacarpal fractures with little angulation, shortening or malrotation, are usually amenable to conservative treatment and very infrequently result in any functional deficit. Fractures with an important initial displacement, however, may require surgical treatment. 1 Percutaneous fixation with Kirschner wires has shown to reduce the potential for stiffness or scarring when compared with open surgery, resulting in higher range of motion scores; this technique does require some postoperative splinting time however. 2 The introduction of intramedullary cannulated screws 3 4 5 represents a minimally invasive, stiffer fixation alternative to the classical elastic pinning methods. Theoretical advantages of a more rigid construct include a diminished need for postoperative splinting, a swifter postoperative period, and a quicker return to work. Early clinical results appear encouraging. Taking into account the important social and economical burden produced by time off work related to this fracture, further studying this promising new fixation method appeared to be extremely relevant. We are unaware of any studies of intramedullary fixation for metacarpal fractures that compare the use of Kirschner wires with intramedullary screws. The aim of this study is to compare both fixation methods in terms of functional results and patient-related outcomes.

## Materials and Methods

The authors performed a systematic review of the hospital's records from 2009 to the present. Patients between 18 and 65 years old, with transverse or short oblique fractures of the neck or shaft, who had been treated either with Kirschner wires (Group A) or intramedullary cannulated screws (Group B) were included. Patients who had sustained other upper extremity injuries or multiple traumas were excluded from the study. Surgical treatment was indicated if there was a rotational deformity of > 5°: for shaft fractures a lateral angulation of > 10° in index and long fingers, 20° for the ring finger, and 30° for the small finger; for fractures of the metacarpal neck with a lateral angulation of 45° or more; and for fractures with complete displacement and no associated injuries. All of the surgeries were conducted by one of the four senior hand surgeons at our institution's had unit.

All of the patients were contacted telephonically and scheduled for a follow-up visit with a fellowship trained hand surgeon at the hospital's clinics. Our institution did not require an institutional review board approval for this study.

### Surgical Technique

Group A: all of the patients were operated with an antegrade Kirschner wire stabilization technique. A small incision was performed proximal to the base of the metacarpal; following subcutaneous dissection, a hole is made through the ulnar or radial cortex of the metacarpal, directed distally to open the canal, avoiding perforation of the contralateral cortex; one or two 1.2 mm Kirschner wires are bent at one end, to control the direction of introduction. The fracture is then reduced and the Kirschner wires are introduced longitudinally, from the metacarpal base up to the metacarpal head. Hardware positioning was controlled intraoperatively with an image intensifier.

**Fig. 1:**
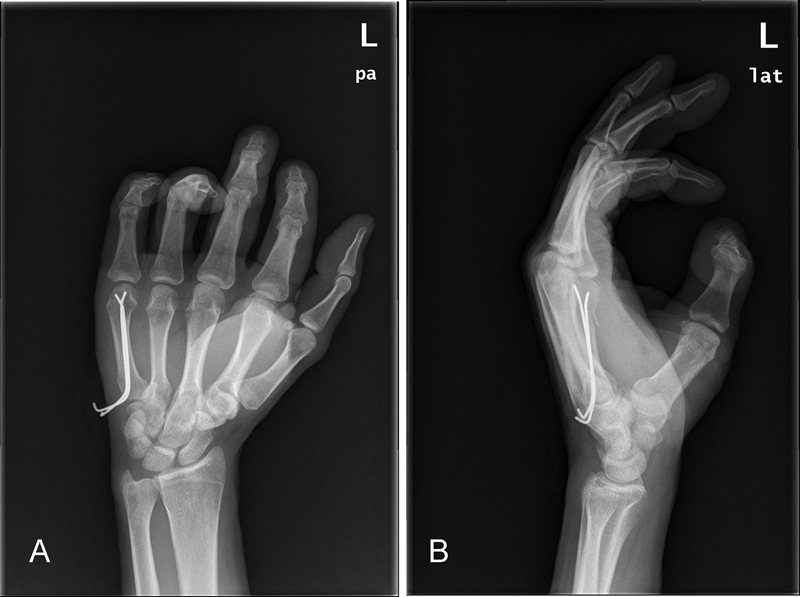
( **A and B** ) Postoperative X-rays of one of our cases treated with antegrade Kirschner wire pinning.

Postoperative X-rays of one of our cases can be seen in Fig. 1A and B .

Group B: a 0.5 cm incision was performed at the level of the metacarpal head, and the extensor tendon was incised at the midline, longitudinally to a similar extent. A 1.0 mm guide wire was inserted along the longitudinal axis of the metacarpal under fluoroscopic guidance. The Kirschner wire was overdrilled and replaced with either a 2.4 or 3.0 mm cannulated headless compression screws, based on preoperative templating. The screw was inserted until all of the distal screw threads surpassed the fracture site. Hardware positioning was controlled with an image intensifier.

**Fig. 2:**
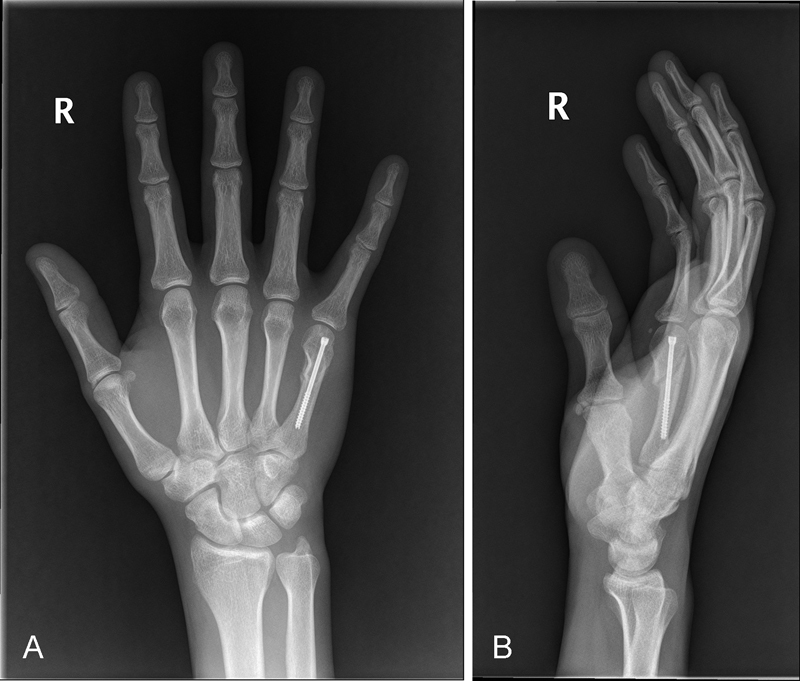
( **A and B** ) Postoperative X-rays of one of our cases treated with an intramedullary cannulated screw.

Postoperative X-rays of one of our cases can be seen in Fig. 2A and B .

### Strength Measurements

Strength measurements were obtained for both groups at the latest follow-up. The mean strength was calculated for every patient following a triplicate measurement with a Jamar dynamometer (Sammons Preston Inc., Bolingbrook, IL) in position two. Approximately 2 minutes were allowed for patient recovery between measurements.

### Range of Motion

Total active range of motion (TAM) was measured at the latest follow-up visit by one of the operating surgeons with a hand-held goniometer. TAM was calculated as the sum of active flexion at the metacarpophalangeal, proximal interphalangeal, and distal interphalangeal joints minus the extension deficits in these joints.

### Patient-Related Outcomes

Patient satisfaction was rated on a scale from 0 (completely dissatisfied) to 10 (completely satisfied); pain was scored with a visual analog scale from 0 (no pain) to 10 (excruciating); all of the patients were asked to fill a validated version of the Quick disabilities of the arm and hand score (DASH) questionnaire at the latest follow-up.

### Statistical Analysis

The data were analyzed with a Mann–Whitney U test, using the SPSS statistical software (IBM, NY).

## Results

A total of 31 patients with 37 metacarpal fractures that were either treated with Kirschner wire fixation or intramedullary screws were identified at our institution's records. One of the patients had sustained a multiple trauma and another one had comminuted fractures, and they were excluded from the study; three other patients were lost to follow-up. This left us with 26 patients and 30 metacarpal fractures. Of these 19 fractures belonged in the screw group and 11 on the Kirschner wire fixation group. There were only three women, all of them in the screw group, and the rest of the patients were men. The mean age was 32.1 years (18–59) for the screw group, and 31.6 for the Kirschner wire group (19–59). There were four second metacarpal fractures, one-third metacarpal fracture, and four-forth metacarpal fractures, and the rest were all fifth metacarpal fractures. All of the patients were right handed except for one, who was in the Kirschner wire group. The right dominant hand was injured in seventeen patients, nine of them in the screw group, and eight in the Kirschner group.

The mean grip strength was 32.6 kg (16–45 kg) in the screw group and 37.3 kg (18–55 kg) for the Kirschner wire group, 88.3% and 89% of the contralateral side respectively; this difference was not statistically significant ( *p* = 0.149).

The mean TAM was 253 (190–285) for the screw group and 265 (212–280) for the Kirschner wire group, 96% and 97.6% of the contralateral side respectively. This was not statistically significant ( *p* = 0.077).

The mean satisfaction was 9.4 (7–10) for the screw group and 9.1 (6–10) for the Kirschner wire group; the mean pain on the visual analog scale was 1 (0–4) for the screw group and 0.8 (0–5) for the Kirschner wire group; and the mean Quick DASH score was 4.7 (0–22.7) for the screw group and 5.2 (0–34.1) for the Kirschner wire group. None of these differences reached statistical significance (the respective *p* values were 0.861, 0.255, and 0.613).

The mean return to work time was 0.92 months for the screw group (0.5–1.5) and 1.86 for the Kirschner wire group (0.1–3); this difference was statistically significant ( *p* = 0.043).

The mean casting time was 4.4 days (0–21) for the screw group and 27.7 days (18–37) for the Kirschner wire group; this difference was statistically significant ( *p* < 0.0001).

All of the fractures were united successfully; we did not register any cases of malunion on either of the groups. One of the patients in the Kirschner wire group referred having experienced an important degree of pain during the removal in clinics; his satisfaction score was 6 out of 10.

Two of the patients, one in the screw group and one in the Kirschner wire group, developed an extension lag, one at the proximal interphalangeal joint and another one at the metacarpophalangeal joint that did not interfere with their activities of daily living; their Quick DASH was 6.8 and 9.1, respectively.

Two of the patients on the screw group developed some degree of stiffness; their respective Quick DASH was 22.7 and 4.5, respectively.

Two of the patients required specific hand therapy, one in the Kirschner wire group and another in the screw group.

None of the patients required a second surgery for implant removal; one of the patients in the Kirschner wire group did not want his hardware removed in clinics; this particular Kirschner wire produced no discomfort to the patient; and it was still in place at the latest follow-up. The two patients who developed stiffness at the screw group did not want any further surgeries done and declined any removal or tenolysis procedures.

Our results are summarized in Tables 1 and 2 .

**Table 1:** Screw group

Patient	Age	Sex	Mtc	Grip	Grip c	TAM	TAM C	C time	RTW	Sat	VAS	Quick DASH
1	21	M	5	35	40	250	260	5	1	9	1	4.5
2	38	M	4	40	48	240	265	5	0.5	10	2	4.5
2	38	M	5	40	48	270	280	5	0.5	10	2	4.5
3	34	F	5	25	28	270	275	6	1	9	1	4.5
4	38	M	5	N/A	N/A	N/A	N/A	0	0.75	10	N/A	N/A
5	24	M	5	61	60	275	275	0	1.5	9	2	2.3
6	59	M	2	20	32	190	230	5	1.5	9	1	4.5
7	19	M	4	28	30	265	265	5	1	10	0	0
7	19	M	5	28	30	285	275	5	1	10	0	0
8	18	M	5	45	48	270	270	0	0.75	10	0	0
9	36	F	4	24	25	275	275	0	0.75	10	0	0
9	36	F	5	24	25	285	285	0	0.75	10	0	0
10	22	M	4	38	40	275	275	0	N/A	10	0	0
11	52	F	2	16	22	200	245	19	1	7	4	22.7
11	52	F	3	16	22	220	240	19	1	7	4	22.7
12	37	M	2	40	50	235	240	21	N/A	10	0	9.1
13	37	M	4	32	34	270	270	0	0.75	8	1	9.1
14	18	M	5	21	22	266	266	0	0.5	10	0	4.5
15	29	M	5	32	38	221	252	0	1	10	2	9.1

Abbreviations: C time, casting time in days; Grip C, contralateral grip strength in kilograms; Grip, grip strength in kilograms; Mtc, Metacarpal; Quick DASH, quick disabilities of the arm and hand score; RTW, return to work in months; Sat, satisfaction; TAM, total active motion; TAM C, contralateral total active motion; VAS, pain on the visual analog scale.

**Table 2:** Kirschner wires group

Patient	Age	Sex	Mtc	Grip	Grip c	TAM	TAM C	C time	RTW	Sat	VAS	Quick DASH
1	19	M	5	37	32	280	280	21	3	6	3	2.3
2	41	M	5	29	33	250	270	18	2.5	10	0	2.3
3	27	M	5	51	41	275	275	21	3	10	0	2.3
4	59	M	5	40	43	275	280	32	0.1	10	0	0
5	29	M	5	55	51	275	275	30	1	9	0	2.3
6	27	M	5	40	40	275	275	30	2	10	0	0
7	20	M	2	46	48	280	280	37	1	10	0	0
8	35	M	5	36	32	280	280	37	2.5	10	0	4.5
9	24	M	5	36	58	275	285	21	1.5	10	0	2.3
10	23	M	5	22	36	242	257	29	2	8	1	6.8
11	44	M	5	18	48	212	233	29	No	8	5	34.1

Abbreviations: C time, casting time in days; Grip C, contralateral grip strength in kilograms; Grip, grip strength in kilograms; Quick DASH, quick disabilities of the arm and hand score; RTW, return to work in months; Sat, satisfaction; TAM, total active motion; TAM C, contralateral total active motion; VAS, pain on the visual analog scale.

## Discussion

The treatment of isolated metacarpal fractures with Kirschner wire pinning has a long and proven track record. This treatment is based on the concept of flexible fixation introduced by Ender and Simon-Weidner in 1970. 6 Foucher 7 described the results of the antegrade pinning technique; on their series of 66 patients with 68 fractures, all of their patients returned to their previous activities; six patients had a 10° extension lag; and other six patients had a 15° extension lag; however, only one patient an auto mechanic complained of this decrease in range of motion.

Intramedullary fixation with headless cannulated screws follows the principles of rigid stable fixation; it allows for early mobilization and decreases the need for postoperative casting.

The first written report of this technique, to the best of the authors knowledge, is a case report by Boulton et al 3 they described the use of the intramedullary headless compression screw technique for the fixation of a fifth metacarpal comminuted neck fracture. The patient's metacarpophalangeal joint flexion at the latest follow-up was 80°; her extension was full. Ruchelsman et al 4 on a 39 patients case series reported a mean 88° metacarpophalangeal flexion, a mean flexion–extension arc of 90°, and a 105% mean grip strength when compared with the contralateral hand. They do not report data for TAM scores, or time off work; their study does not include a control group.

del Piñal et al 5 published a 63 fractures case series; they do report TAM scores, and time off work, but not for grip strength. Their case series is heterogeneous; it includes phalanx and metacarpal fractures of different types.

Our review included transverse or oblique fractures of the metacarpal neck or shaft, and we had a control group with similar fractures treated with antegrade Kirschner wire pinning.

On our case series, there were no differences between the two in terms of ranges of motion, grip strength, satisfaction, postoperative pain, or Quick DASH score.

We did find differences in terms of postoperative splinting time; this was not surprising, as we only applied splinting for a very brief time on some of the patients on the screw group for comfort purposes. The mean return to work time or time back to their regular activities appeared to be shorter on the screw group.

Our study did have some limitations. It was retrospective in nature, and the patient number was limited. Small differences in some of the outcome parameters may not be detectable. In addition, four different surgeons performed the surgeries.

On this case series, we found no differences in terms of function or patient-related outcomes, between the two techniques. The screws appeared to require less casting and provide a quicker return to work. Due to the small number of cases, however, we have been unable to conclude that there were any benefits in the application of one particular technique when compared with the other. The use of cannulated screws must be carefully weighed by the surgeon. The potential downsides, include higher implant costs, the production of an injury to the articular cartilage and the retention of metallic hardware.
